# The Pneumococcus Carrier

**Published:** 1915-09-11

**Authors:** 


### The Pneumococcus Carrier.

To those morbid conditions which have been proved to be spread by means of human carriers, pulmonary infection with the pneumococcus and pneumobacillus must now be added. The increased experience gained in connection with the dispensary treatment of pulmonary tuberculosis shows that in a certain percentage of cases having definite physical signs in the chest and applying for sanatorium benefit, the morbid condition has as its immediate causative agent the pneumococcus; and in some cases the pneumobacillus. Chronic pneumococcal infection of the lungs may date from a well-defined attack of lobar pneumonia, or it may be superimposed on a latent long-standing tuberculous lesion, and be of indefinite duration. These two definite types of cases—namely, the post-pneumonic and the tuberculous—appear to be both the most frequent type of carrier and the most frequent cause of the spread of the disease by direct infection. In children, also, pneumococcal infection of the lungs is by no means infrequently met with, although the child as a carrier of the pneumococcus is probably a much less serious menace than the ignorant adult. The tendency of lobar pneumonia to recur in the same individual is well known, but whether a second attack is due to an auto- or hetero-infection it is impossible to say; but generally the pneumococcal carrier may be regarded as a greater danger to contacts than to himself. Recently the writer had under observation the case of a married woman who had a chronic pneumococcal infection apparently superimposed upon an old tuberculous focus. Within the short space of two months the father and three sons were attacked with acute lobar pneumonia, the disease in the case of the father and one son proving fatal. The third son came under the writer's observation some weeks after he had recovered from pneumonia; physical signs persisted, and pneumococci in considerable numbers were present in the sputum. Other cases of pneumococci carriers have been under observation, and it is obvious that such present a definite source of danger which calls for clearly defined preventive measures. These should include the more frequent examination of the sputum for the presence of pneumococci in all chest cases from which tubercle bacilli are absent, and the notification of all cases of pneumococcal infection. In chronic cases special precautions with regard to the disposal and destruction of the expectoration are called for. Lastly, active treatment is indicated in the case of all pneumococcal carriers. This includes antiseptic inhalations, the administration of creosote or guaiacol, and injections of pneumococcal vaccine.

